# A Vignette-Based Measure of Mental Health Literacy (PDR-V): Reliability, Validity, and Mindfulness Associations in a Cross-Sectional Sample

**DOI:** 10.3390/ijerph23010031

**Published:** 2025-12-24

**Authors:** Matea Gerbeza, Saba Salimuddin, Jenna Kazeil, Shadi Beshai

**Affiliations:** 1 Department of Psychology, University of Regina, Regina, SK S4S 0A2, Canada; 2 Department of Psychological Science, Kennesaw State University, Kennesaw, GA 30144, USA

**Keywords:** mental health literacy, dispositional mindfulness, mindfulness, mental health, treatment seeking, treatment seeking attitudes, vignette methodology

## Abstract

Psychological distress impacts a large portion of the general population. While effective treatments are available, few seek them out. This lack of treatment seeking may be due to several factors, particularly low mental health literacy (MHL). MHL is the knowledge an individual has regarding psychological disorders and their symptoms, treatments, and where to seek appropriate help when identified. The capacity to pay attention to present-moment experiences in MHL translates to the qualities of dispositional mindfulness (DM), the capacity to pay non-judgmental attention to present-moment experiences. The purpose of the present study was to evaluate the reliability and preliminary convergent validity of a newly developed, vignette-based assessment of psychological disorder recognition. A total of *N* = 299 participants were recruited via TurkPrime and completed measures of DM (FFMQ), MHL (MHLS), depression (PHQ-9), anxiety (GAD-7), and treatment-seeking attitudes (MHSAS). Participants were subsequently asked to read newly created vignettes based on ICD-11 criteria of major depressive, generalized anxiety, social anxiety, bipolar disorders, post-traumatic stress disorder, and schizophrenia. Participants then responded to questions assessing the recognition of disorder presence and identification. The vignettes with accompanying questions were titled the Psychological Disorder Recognition—Vignette (PDR-V) task. The PDR-V evidenced a Kuder–Richardson Formula 20 (KR-20) of 0.83, indicating excellent internal consistency. Independent sample *t*-tests indicated that participants with prior psychotherapy exposure, a history of mental health diagnosis, and, unexpectedly, those reporting lower education levels and no current mindfulness practice, scored significantly higher on the PDR-V. Spearman correlations revealed that higher scores on a validated MHL scale and specific facets of DM (describe, act with awareness) were positively correlated with PDR-V scores. Bipolar disorder evidenced the highest recognition as a psychological problem broadly, while social anxiety had the highest specific disorder identification accuracy rates. Generalized anxiety disorder had the lowest recognition and identification accuracy. While the PDR-V demonstrated promising preliminary psychometric properties, it also observed anomalies that warrant further investigation, as findings are limited by its cross-sectional nature. These findings suggest that the PDR-V is a versatile tool for differentiating the presence of a problem and accurately identifying the condition, supporting its potential as a reliable and sound measure.

## 1. Introduction

In 2019, the World Health Organization [1] indicated that 970 million people around the globe were living with a psychological disorder. Those with severe psychological conditions were also reported to die approximately 10 to 20 years earlier than those without such conditions within the general population [1]. Despite these alarmingly high rates, access to treatment for psychological distress varies. Depression impacts approximately 3.8% of the global population, while 4% experience an anxiety disorder [1]. Only 47% of those experiencing psychological disorders in North America are able to access appropriate treatments [2]. This may be due to several factors, including feasibility, stigma, and lack of appropriate guidance and awareness on where and how to receive help [3]. Without appropriate treatments, psychological disorder symptoms may be exacerbated, as unaddressed symptoms are associated with deleterious outcomes such as poverty, social isolation, chronic illnesses, and suicide [4]. The recognition of mental distress and knowledge about appropriate ways to manage it are major components of mental health literacy (MHL) [5].

MHL is defined as the knowledge, beliefs, and attitudes that an individual has about psychological disorders and distress that assist in their recognition, management, and prevention [5]. MHL is composed of six core components: (a) the ability to recognize specific disorders or different types of psychological distress; (b) knowledge and beliefs about risk factors and causes; (c) knowledge and beliefs about self-help interventions; (d) knowledge and beliefs about professional help available; (e) attitudes that facilitate recognition and help-seeking; and (f) knowledge about how to seek mental health information [5]. In the context of MHL research, much of it relies on self-report questionnaires in order to broadly examine MHL components.

MHL is highly relevant within public health efforts to decrease rates of psychological disorders. Researchers have found that barriers to treatment included lack of knowledge about where to receive treatment (24%), the belief that one can handle the problem without treatment or that treatment would not be helpful (37%), fear of involuntary hospitalization or taking medications (12%), and heightened stigma (10%) [3]. Increasing MHL may address many of these barriers. This is because the nature of MHL is to seek and internalize information on where to seek appropriate treatment, appropriate knowledge on self-help interventions, and increasing positive attitudes about seeking treatment [5]. A digital app was recently designed as a MHL campaign for adolescents, focusing on competition, social media, perfectionism, loneliness, isolation, and independence [6]. The results supported the app’s usage as a tool to increase MHL, as the results demonstrated increases in mental health knowledge, willingness, and confidence to seek treatment, as well as reduced stigma towards depression [6].

There are several individual-difference factors, such as demographic factors, which may reliably predict MHL. For example, factors such as employment and marital status are associated with treatment seeking, while education level is associated with MHL [7]. Additionally, in a recent study, researchers found that high performers on self-reported MHL measures tended to be female and between the ages of 28 and 32 years [8]. Relatedly, health literacy, one’s knowledge regarding physical health information and services, has shown associations with several demographic variables [9]. Lower health literacy was associated with older age, lower educational level, and lower income [9]. Extrapolating from the associations of demographic variables within the context of general health literacy, it is plausible to assume that such demographic factors correlate meaningfully with MHL.

Awareness and labelling of internal distress are important aspects of MHL. Accordingly, cultivations of these qualities may aid in reducing distress in the long-term. Pertinently, mindfulness—a construct that originated from Buddhism and other Eastern contemplative traditions—centers on awareness [10]. Currently, there is no consensus on mindfulness’s theoretical framework and its effects on mental health; however, most researchers believe the construct is composed primarily of attention regulation and the attitudinal qualities of acceptance, openness, and curiosity [11]. Mindfulness is typically defined as paying attention to the full gamut of present-moment experiences with openness, curiosity, and acceptance [10,12].

Mindfulness as a concept can manifest as either a state or a disposition [13,14]. State mindfulness refers to paying momentary attention with the qualities of mindfulness in specific situations, whereas dispositional mindfulness (DM) refers to one’s tendency or natural (uncultivated) capacity to use mindfulness across situations [14]. DM’s main feature is consistent with the broader construct of mindfulness, as it is the natural ability to have “present-centered attention”, without the conscious effort to engage in mindfulness practices as seen in state mindfulness [15]. DM is often theorized to be multifaceted [11]. For example, Baer et al. (2006) [16] evaluated mindfulness questionnaires and proposed five facets. These facets include observe, or attending to thoughts, emotions, and sensations; describe, or the capacity to label internal experiences; acting with awareness, or the full engagement in activities rather than habitual responding; non-judgment, or the capacity to experience without judgment; and non-reactivity, or the capacity of “letting go” and nonattachment to thoughts and emotions [16]. Other researchers frame DM as requiring the qualities of patience, loving kindness, the ethical principle of doing no harm, and cultivating knowledge [17]. Accordingly, there are several conceptualizations of DM, and this heterogeneity in definitions has spilled over into how the construct is measured.

Despite the inconsistency in DM definitions, mindfulness as a broad construct is still universally emphasized as embracing non-judgmental awareness of internal and external stimuli [10]. Accordingly, there is a plausible theoretical relationship between DM and MHL. Facets that comprise mindfulness in Baer and colleagues’ (2006) [16] framework lend support for this notion, as describe, acting with awareness, and non-judgment were inversely related to alexithymia, a construct that defines a person’s difficulty in recognizing and describing emotions [18]. Lo et al. (2023) [19] demonstrated that mindfulness was a significant mediator between social support and MHL, providing evidence for the relationship between DM and MHL. In a systematic review, researchers stated that when mindfulness exercises have been implemented, symptoms of anxiety and depression were decreased through increasing awareness, skills, and efficiency, which are all critical components of MHL [20].

DM was previously assessed for its relationship with MHL and treatment-seeking attitudes (TSAs) [21]. The authors examined whether any of the FFMQ facets predicted and moderated MHL and TSA [21]. The results indicated that significant predictors of MHL included describe and non-reactivity, while act with awareness predicted TSA [21]. Non-reactivity also served as a significant moderator between MHL and TSAs [21]. The Theory of Planned Behaviour (TPB), a concept that highlights an individual’s attitudes, norms, perceived behavioural control, and personal intentions to predict their behaviour [22], was hypothesized to explain this relationship noted between MHL, TSAs, and DM facets [21]. DM may be connected with attitudes, subjective norms, and perceived control over psychological distress [21]. Additional evidence included a study where DM moderated the relationship between self-control and psychological distress [23]. It is possible that there is a relationship between MHL and DM, specifically in earlier treatment initiation, through TPB components, but it can only be assessed further with proper measures.

In addition to individual differences, the methodology employed is also a critical aspect of understanding MHL. Self-report measures of MHL are advantageous in many ways, such as having superior time and cost-efficiency [24]; however, there are limitations with this approach. Self-report scales more aptly measure what individuals *believe* they know or understand, rather than how well they can apply that knowledge to specific situations or contexts [25]. In early MHL research, disorder recognition was often examined using vignettes or short stories that describe a fictional patient experiencing psychological disorder symptoms, followed by an assessment of the participant’s ability to accurately identify the disorder depicted in the vignettes [26,27]. These vignette-based measures are considered more performance-based, as they require individuals to apply their current knowledge to concrete clinical presentations rather than endorsing general statements, with the latter being associated with increased likelihood of bias [28,29]. Using the vignette-based methodology, Jorm and colleagues [27] found that 72% of participants accurately recognized symptoms of depression, and 84% were able to accurately identify schizophrenia. In an Australian study, researchers found that only 68% of rural, adolescent participants could identify depressive symptoms [30]. Accordingly, while self-report measures are dominant in MHL research, other methodologies, such as vignette-based approaches, have also been used and may offer more ecological validity in examining disorder recognition.

However, the vignette approach in MHL research has historically faced two key limitations. First, researchers have often relied on the recognition of only one or two vignettes, rather than providing participants with a broader range of case studies to assess their ability to detect diverse disorders with variable presentations. Second, these approaches have frequently conflated the general presence of a problem with specific diagnostic recognition. This is a critical oversight, as Jorm’s foundational definition of MHL explicitly calls for parsing out these related but distinct factors.

## 2. Current Study

An individual’s ability to accurately label emotional and cognitive experiences is highly relevant to the identification of clinically significant psychological distress symptoms. One’s willingness to consider that a repetitive pattern of thoughts, emotions, and behaviours may be consistent with a psychological condition, instead of dismissing these symptoms as only reactions to stress. Evidently, this suggests that having higher DM may be related to higher MHL, specifically accuracy in psychological distress symptom recognition and its connection to a possible psychological condition.

Despite vignette research demonstrating strength in MHL research, vignette studies typically do not report full psychometric detail, such as item difficulty, internal consistency, and confusion patterns (i.e., which psychological disorders are mistaken for which). The current study addresses these critical gaps by treating vignette-based recognition as a measurable construct (psychological disorder recognition vignette task; PDR-V), rather than a one-off outcome. The objectives of this study were to (1) separate problem detection from diagnostic identification via a two-step structure (Question 1: Is the problem psychological versus physical/no problem/other?; Question 2: If deemed psychological, which diagnosis?); (2) include a non-psychological decoy to indicate specificity (over-/under-pathologizing) without contaminating scores; and (3) report item-level performance to address difficulty level (corrected item-total correlations), internal consistency (KR-20 for binary items), and confusion matrices for diagnostic options. In terms of conception, we aimed to evaluate convergent and discriminant patterns with mindfulness facets (i.e., observe, describe, non-judgment, non-reactivity, and act with awareness), with a priori emphasis on describe, one’s ability to label internal experiences. Finally, we examined the relationships of PDR-V to established MHL and TSAs measures. We position PDR-V as a recognition-domain indicator within MHL using a general adult sample from the first author’s honours thesis [31].

Hypotheses for the present study were the following. Firstly, H1 hypothesized that the PDR-V measure would showcase convergent validity through a significant and positive correlation with the validated Mental Health Literacy Scale (MHLS), which is designed to capture a respondent’s MHL levels across several domains. Detection and labelling of disorders on the PDR-V should be predicted by having higher literacy scores on the MHLS. Secondly, we hypothesized H2. Based on the premise that MHL requires non-judgmental attention to present-moment experiences, it was hypothesized that higher levels of DM (specifically the describe facet of the FFMQ) would be positively associated with PDR-V recognition scores. We also hypothesized in H3 that participants with lived experiences of mental health interventions (e.g., prior exposure to therapy, prior mental health diagnosis) would have higher PDR-V scores. Fourth, we hypothesized in H4 that current psychological distress (elevated PHQ-9 and GAD-7) would be negatively correlated with PDR-V sores. Finally, we examined differences in PDR-V based on known differences in demographics and their relationship with MHL.

## 3. Method

### 3.1. Participants

Respondents were recruited online from five English-speaking nations, Australia, Canada, New Zealand, the United Kingdom, and the United States, using TurkPrime, which is an extension of Amazon Mechanical Turk (AMT). TurkPrime is a crowdsourcing website providing individuals the opportunity to participate in research studies for modest monetary compensation [32]. Participants in the present study were compensated 2.50 USD, which is commensurate with other crowdsourcing studies [33,34]. The original sample consisted of 313 respondents. Due to low English language proficiency or not answering the English language proficiency item, *n* = 12 were excluded. Two participants were excluded for failing our attention check question. For the independent sample *t*-tests, sample size differed mildly due to binary outcomes being used (e.g., “prefer not to disclose”; see Section 3.4). For prior psychotherapy exposure, *n =* 287 was used, and for prior mental health diagnosis, *n* = 286 was used. Current mindfulness practices utilized *n* = 295, and gender consisted of *n* = 298. All other analyses utilized *n* = 299. Ethics was granted by the first author’s institutional research ethics board.

### 3.2. Procedure

Potential respondents viewed the study title and a brief description online through TurkPrime. If they were interested in participating, they were asked to click the hyperlink that would take them to the study’s survey. The survey was hosted on the survey platform Qualtrics. Once individuals provided their informed consent, they proceeded to respond to study measures. Following completion of these measures, participants were presented with eight short case vignettes. Each vignette was followed by two questions assessing recognition and disorder identification of the presenting problem within the vignette. Participants subsequently completed the Mental Health Literacy Scale (MHLS-35) [35]. All measures, except for the MHLS-35, were presented in a randomized order. The MHLS-35 was not randomized, as it was deemed to be logical to present it at the very end, as some of the items may introduce biases if it were presented before the vignettes (e.g., “how willing would you be to move next door to someone with a mental illness?”). Lastly, participants completed a demographic information form assessing pertinent demographics, which included an attention check item (i.e., “What was this survey about? Please do not select “Mindfulness” but instead choose “Other” and type “Psychology” in the textbox”) to screen out those who were potentially bots or mindlessly responding. A debriefing form was provided, and AMT automatically compensated participants.

### 3.3. Measures

#### 3.3.1. Patient Health Questionnaire-9

The Patient Health Questionnaire-9 (PHQ-9) [36] is a 9-item self-report measure of depressive symptoms consistent with the Diagnostic and Statistical Manual for Mental Disorders (DSM) [37] over the past two weeks. Participants are prompted with the phrase “over the last two weeks, how often have you been bothered by any of the following problems”, and their responses are rated on a 4-point Likert scale that ranges from zero, or “not at all”, to three, or “nearly everyday”. Higher scores on the PHQ-9 indicate more severe depressive symptoms. In the present study, Cronbach’s alpha and McDonald’s omega were both 0.92, demonstrating strong reliability. The *M* was 8.02 and SD was 6.87.

#### 3.3.2. Generalized Anxiety Disorder-7

The Generalized Anxiety Disorder-7 (GAD-7) [38] is a 7-item self-report measure that examines symptoms of generalized anxiety and is consistent with the DSM [37] over the past two weeks. Participants are presented with the prompt “over the last two weeks, how often have you been bothered by any of the following problems”, and responses are rated on a 4-point Likert scale ranging from zero, or “not at all”, to three, or “nearly everyday”. Higher scores on the GAD-7 are indicative of more severe generalized anxiety symptoms. In the present study, Cronbach’s alpha and McDonald’s omega were 0.93 and 0.94, respectively, indicating strong reliability. The *M* = 6.62 and SD = 5.84.

#### 3.3.3. Five Facet Mindfulness Questionnaire-24

The Five Facet Mindfulness Questionnaire-24 (FFMQ-24) [39] is a 24-item self-report measure. It is a brief version of the 39-item original scale [16], and it assesses an individual’s DM on five individual factors: observe, describe, act with awareness, non-judgment, and non-reactivity. It is measured on a 5-point Likert scale, ranging from one (never or very rarely true) to five (very often or always true). Higher scores are indicative of higher DM levels once negatively keyed items are reverse-scored. For the current study, Cronbach’s alpha was 0.90, and McDonald’s omega was 0.88, with *M* = 82.56, and SD = 14.50.

#### 3.3.4. Mental Health Literacy Scale-35

The Mental Health Literacy Scale-35 (MHLS-35) [35] is a 35-item self-report questionnaire that examines an individual’s knowledge and understanding of different areas of mental health. There are several Likert-type formats for items. For items 1 to 10 and 13 to 15, responses are rated on a 4-point Likert scale (1 = very unlikely to 4 = very likely); for items 11 and 12, responses also use a 4-point format, but their qualitative description differs, as it ranges from 1 (very unhelpful) to 4 (very helpful). For items 16 to 28, a 5-point Likert format is used (1 = strongly disagree to 5 = strongly agree). For items 29 to 35, a 5-point Likert scale is also used, but qualitative description differs (1 = definitely unwilling to 5 = definitely willing). Higher total scores are reflective of better knowledge of mental health concepts. Cronbach’s alpha and McDonald’s omega for the present sample were evaluated separately for both the 4- and 5-point response scales. Cronbach’s alpha was 0.86 for the 4-point Likert scale items and 0.89 for the 5-point Likert scale items. McDonald’s omega for the 4-point Likert scale was 0.86, and for the 5-point Likert scale, it was 0.88. The *M* = 123.09 and SD = 16.34.

#### 3.3.5. Mental Help-Seeking Attitudes Scale-9

The Mental Help-Seeking Attitudes Scale-9 (MHSAS-9) [40] is a 9-item scale rated on a 7-point semantic differential scale and developed in order to measure respondents’ overall evaluation of mental health professionals when deciding whether to seek help for experiences of psychological distress. Participants are prompted with the following phrase, “If I had a mental health concern, seeking help from a mental health professional would be…”, and asked to select a response that best displays their attitudes from pairs of opposite descriptors (e.g., useful vs. useless). Items two, five, six, eight, and nine are reverse-scored. Responses are scored from 1 to 7 for each adjective pair, with participants selecting only one option per item. Scores range from 9 to 63, with valence of item anchors counterbalanced across the items by having positively valenced items on both left and right sides of the scale. For example, “useful” was on the right side, and “important” was on the left side, with their opposites on the other side of the scale. Participants scoring higher on the MHSAS indicate more positive attitudes towards seeking help for psychological distress. Cronbach’s alpha for the present study was 0.94, and it was 0.94 for McDonald’s omega, with *M* = 48.39 and SD = 11.39.

#### 3.3.6. Psychological Disorder Recognition—Case Vignettes

Psychological Disorder Recognition Vignettes (PDR-V; developed by the first, second, and last authors) were developed and presented to participants as an additional, potentially more ecologically valid assessment of the symptom recognition aspect of MHL. Participants were presented with eight vignettes describing fictional cases of patients reporting symptoms of either a psychological or physical condition: major depressive disorder (MDD), social anxiety disorder (SAD), generalized anxiety disorder (GAD), post-traumatic stress disorder (PTSD), bipolar disorder, schizophrenia, asthma, and heart disease. All vignettes have been included in a Supplemental File (Supplement S1). After reading each case vignette, participants were asked to answer the following questions to assess their psychological disorder recognition and identification accuracy:

Question 1: “Is the individual in the above case sample experiencing a psychological, physical, or other type of problem?” (options: psychological, physical, other).

Question 2: “What disorder or problem is the individual experiencing? If other, please specify” (options: MDD, GAD, SAD, bipolar, PTSD, schizophrenia, asthma, heart disease, cancer, diabetes, stroke, arthritis, other).

The case vignettes were developed in accordance with the International Classification of Diseases and Related Health Problems [41] criteria to account for the international sample, with consensus about their content being obtained from three mental health researchers. Stimulus persons were named according to Newman et al.’s (2018) [42] paper on assigning names in case vignettes. Names perceived as high warmth and high competence were selected. The ages of stimulus persons were chosen through a random number generator, with ages ranging from 30 to 55 years, as this is the age range that best represents the age of onset of the mental disorders being described within our vignettes [43]. The MDD, GAD, and asthma case vignettes were created in the context of another trial for a Master’s thesis [44]. The remaining five vignettes were created for the purpose of this study and served as a template. The heart disease vignette was adapted from Wijeratne & Harris’ (2009) [45] paper but was modified to include a name consistent with Newman et al.’s (2018) [42] suggestions, as well as altering the age to remain consistent with the ages in the other case vignettes. Asthma and heart disease vignettes were included to prevent response biases in our participants.

For the psychological vignettes, Question 1 was scored “1” if “psychological” was selected and “0” if physical or other was selected. Question 2 was scored “1” if the keyed diagnosis for that vignette was selected or “0” if the incorrect diagnosis was selected. Vignette scores ranged from 0 to 2 (Question 1 + Question 2). Scores across psychological disorder vignettes were summed to create a PDR-V total (maximum score of 2 multiplied by 6 psychological disorder vignettes for a maximum total score of 12), and a proportion correct index was computed (total divided by 2 multiplied by *N* psychological disorder vignettes). The physical disorder decoy vignettes were not included in the scoring and remained unscored, but their Question 1 accuracy (identifying the problem as physical/other + heart disease/asthma) is reported descriptively as a specificity and quality check. Higher scores indicated greater psychological disorder recognition and identification. This two-step structure separates problem detection/domain (Question 1) from diagnostic identification (Question 2), allowing error-type documentation (i.e., false negatives and domain errors versus label errors) and a specificity check via the decoy. Cronbach’s alpha for the vignettes was calculated to be 0.85, and McDonald’s omega was 0.86, with *M* = 10.04 and SD = 2.57.

### 3.4. Data Analyses

Data was reviewed for completion and missing data and was cleaned and reverse-scored where appropriate. After applying our inclusion criteria (18+, having an English language proficiency of at least 6/10, and passing our attention check question) and having additional parameters for being excluded in the analyses, including participants with less than 80% of their data, there was no missing data. PDR-V internal consistency was evaluated using Kuder–Richardson Formula 20 (KR-20), an equivalent tool to Cronbach’s alpha for binary items [46]. We also report item difficulty, specifically the proportion of participants who answered each vignette correctly, corrected item-total correlations, and a confusion matrix (true diagnosis × selected diagnosis) to identify misclassifications. These indices are presented as evidence of reliability and preliminary validity for the PDR-V as an index of recognition-based MHL.

For hypotheses one through four, Spearman correlations were run between scores on the PDR-V and FFMQ facets (i.e., observe, describe, act with awareness, non-reactivity, and non-judgment) [16,38]. We anticipated that the facet of describe would demonstrate the strongest relationship with PDR-V due to the theoretical argument that the ability to label internal experiences is relevant to symptom recognition, and the facet of describe focuses on the labelling of one’s experiences [16]. These correlations provide concurrent validity that is specific to symptom recognition, as it aligns with previously reported associations between FFMQ’s facets and MHL [21].

Additional independent samples *t*-test analyses were conducted to examine how PDR-V scores differed across pertinent demographics and lived experience components. We compared recognition scores across education levels as an external validity check, as MHL tends to be higher in samples with more formal education [47,48]. Education levels were re-coded to become binary variables, with those having anything less than a Bachelor’s degree being collapsed into one group and those with a Bachelor’s degree or higher being collapsed into another, with years of education being the basis for separating groups in order to run an independent samples *t*-test. Additionally, a median split was computed for income in order to examine MHL across two groups: those who fell at or below the median were grouped, and those above the median were grouped. Research demonstrates that higher socioeconomic status often co-occurs with more utilization of psychological services [49].

Participants who have had prior psychotherapy, a mental health diagnosis, or engage in a current mindfulness meditation practice may inadvertently have more direct contact with diagnostic language, psychoeducation, treatment concepts, and mindfulness experience, which should translate to better recognition of internal experiences. Therefore, prior psychotherapy exposure and mental health diagnoses, and those currently engaging in a mindfulness practice, were collapsed into those who had responded “yes” and those who responded with “no” in order to run three independent sample *t*-tests. For those who responded with “prefer not to disclose”, they were not included in the data analyses for prior psychotherapy exposure, prior mental health diagnosis, and current mindfulness meditation practices, as the analyses required binary variables. Gender was also considered and was collapsed with those responding as “cis-man”, “man”, “male”, or “trans man” into “man”, and those responding as “cis-woman”, “woman”, “female”, or “trans woman” into woman. Participants who preferred not to disclose were excluded from the analyses as they were too small a sample for inferential testing. However, specificity analyses were conducted and are available in Supplement S2.

PDR-V group differences were evaluated using independent samples *t*-tests for binary factors (education, income, psychotherapy exposure, history of mental health diagnosis, current mindfulness-based practice, and gender). We report means, standard deviations, test statistics, 95% CIs, and adjusted *p*-values within each family of comparisons using Holm–Bonferroni corrections.

## 4. Results

### 4.1. Descriptives

A total of *N* = 313 completed the survey; however, the final sample used in data analyses was *N* = 299 participants (*n* = 287 for prior psychotherapy exposure, *n* = 286 for prior mental health diagnosis, *n* = for 295 for current mindfulness meditation practices, and *n* = 298 for gender independent sample *t*-tests; see the respective data analysis sections) after applying inclusion criteria. The data was skewed, and, therefore, Mann–Whitney tests were computed, but they found no differences between the independent sample *t*-tests, and, therefore, independent sample *t*-tests are reported. Pertinent participant demographics are reported in Table 1. Education collapsed to become binary variables: those below a Bachelor’s degree *(n* = 121) and those with a Bachelor’s degree or higher (*n* = 178). The income median was 31,000 to 50,000 CAD; therefore, those at this income level and below were grouped into one variable, and those above were grouped in another. Those who responded “other” *(n* = 3) were excluded from the income analyses. Means, standard deviations, KR-20s, range of difficulty, and corrected item-total correlations for the PDR-V total, question one, and two scores are presented in Table 2. Table 3a presents the confusion matrix for question one (i.e., whether it is a psychological, physical, or other problem being experienced) and question two (i.e., what disorder is being experienced). The confusion matrix demonstrates that there may be concept overlap between these disorders. Problem recognition scores are presented in Table 4 as means, standard deviations, proportion correct for each question per psychological disorder vignette, the PDR-V overall score, and frequencies for total correct out of 12.

### 4.2. Spearman Correlations

A total of nine Spearman correlations were conducted for PDR-V with FFMQ’s facets of describe, observe, act with awareness, non-judgment, and non-reactivity, as well as depressive (PHQ-9), anxiety (GAD-7), mental help-seeking attitudes (MHSAS), and MHL (MHLS) scores. Correlation coefficients are presented in Table 5. Due to multiple analyses being run, Holm–Bonferroni corrections were conducted to control for type one error. Uncorrected significant correlations are reported for secondary analyses (e.g., inter-facet correlations such as describe and observe) and for associations that did not retain significance following Holm’s correction (e.g., non-reactivity). PDR-V demonstrated the strongest association with the facets of describe (*p* < 0.001; pHolm < 0.05; 95% CI [0.086, 0.292]) and act with awareness (*p* < 0.001; pHolm < 0.05; 95% CI [0.083, 0.330]), as well as the MHLS (*p* < 0.001; pHolm < 0.05; 95% CI [0.454, 0.626]), followed by PHQ-9 scores (*p* = 0.002; pHolm < 0.05; 95% CI [−0.289,−0.059]). No other facet, GAD, or MHSAS scores showed significance or survived the Holm–Bonferroni correction. These results suggest that PDR-V may be associated with processes related to accurate identification and recognition of psychological distress and overall MHL.

### 4.3. Independent Sample t-Tests

We conducted six independent sample *t*-tests, each with a Holm–Bonferroni correction applied. All *t*-tests resulted in significant Levene’s Test for Equality of Differences, except for gender, and, therefore, Welch’s *t*-test results are reported (statistics are reported in Table 6). There were significant differences between the two education groups with *t*(280.327) = 6.345, *p* < 0.001, pHolm < 0.05, *d* = 0.67, and 95% CI [0.432, 0.906], as those with an education below a Bachelor’s degree scored significantly higher on PDR-V (*M* = 11.02, *SD* = 1.51) than those with an education at the Bachelor’s level or higher (*M* = 9.38, *SD* = 2.91). Prior psychotherapy exposure yielded significant group differences, *t*(225.122) = −5.445, *p* < 0.001, pHolm < 0.05, *d* = −0.591, and 95% CI [−0.853, −0.328], demonstrating that those with prior psychotherapy experience scored significantly higher for total PDR-V (*M* = 11.09, *SD* = 1.74) than those without psychotherapy experience (*M* = 9.60, *SD* = 2.75). Similarly, having a prior mental health diagnosis (*M* = 10.91, *SD* = 1.79) compared to not having one (*M* = 9.61, *SD* = 2.79) yielded group differences, with *t*(263.873) = −4.751, *p* < 0.001, pHolm < 0.05, *d* = −0.518, and 95% CI [−0.768, −0.267]. These group differences indicate that participants with a prior mental health diagnosis scored significantly better on the PDR-V than those without. Further differences were noted between those with a current mindfulness meditation practice and those without, with *t*(118.546) = 3.854, *p* < 0.001, pHolm < 0.05, *d* = 0.574, and 95% CI [0.318, 0.830]. This demonstrates that participants without a current mindfulness meditation practice scored significantly higher on the PDR-V (*M* = 10.45, *SD* = 2.18) than those with a current mindfulness meditation practice (*M* = 9.02, *SD* = 3.12). No significant differences were noted between those with an income of 50,000 CAD or below or 51,000 CAD or higher, with *t*(254.242) = 2.161, *p* = 0.032, pHolm < 0.05, *d* = 0.258, and 95% CI [0.027, 0.487]. Those with an income of 50,000 CAD or lower did not score significantly different on the PDR-V (*M* = 10.36, *SD* = 2.31) compared to those with an income of 51,000 CAD or higher (*M* = 9.70, *SD* = 2.81). No significant differences were noted for those identifying as a woman (*M* = 10.11, *SD* = 2.72) or a man (*M* = 9.97, *SD* = 2.42), with *t*(296) = 0.493, *p* = 0.622, pHolm < 0.05, *d* = 0.057, and 95% CI [−0.170, 0.284], on the PDR-V. Specificity analyses were conducted (Supplement S2) and indicated no significant differences among participants who identified as any other gender than man or woman, who were included and excluded from the *t*-test (i.e., trans man, open response).

### 4.4. PDR-V Psychometrics and Recognition Rates

The 12-item PDR-V demonstrated strong internal consistency, as demonstrated by KR-20 = 0.83. Item difficulty ranged from 0.72 to 0.95, and corrected item-total correlations ranged from 0.32 to 0.72. Table 2 presents all means, standard deviations, K-20s, range of item difficulty, and corrected item-total correlations for PDR-V totals, and each disorder is examined individually and by question.

Recognition scores for psychological disorders (e.g., MDD, GAD, SAD, PTSD, schizophrenia, bipolar disorder) were tabulated by completing two questions that corresponded to their vignette, identifying first whether it was a physical or psychological problem and then accurately identifying the psychological disorder that was being presented. Overall, problem recognition rates were high, with *M* = 10.04 out of 12, and *SD* = 2.57. Bipolar disorder had the highest recognition rate of being a psychological disorder, with 95% of the sample correctly recognizing it as a psychological disorder, with schizophrenia and SAD following closely behind, with recognition rates of 94%. Correct labelling of psychological disorders did not follow a similar trend, with 82.9% correctly labelling SAD and 79.6% correctly labelling PTSD and MDD, with schizophrenia (79.3%) and bipolar (78.3%) following closely behind. GAD had the lowest recognition rate of being a psychological disorder (74.2%) and correct labelling (72.2%).

For both questions one and two, the most difficult disorder was GAD, with 0.74 proportion correct, and it was 0.72 for question two. Bipolar disorder was the most common correct disorder for question one (0.95 proportion correct), and SAD was the most common correct disorder for question two (0.83 proportion correct). Table 4 details problem recognition scores for each disorder alone and then altogether.

Though not computed as a total score or included in any analyses, the physical condition decoy (i.e., asthma and heart disease) frequencies are reported, with a total of 91.3% (*n* = 273) correctly classifying asthma as a physical health condition and 82.9% (*n* = 248) correctly identifying it as asthma. For heart disease, 96% (*n* = 287) correctly selected it as a physical health problem, and 75.9% (*n* = 227) correctly identified it as heart disease.

## 5. Discussion

The present study contributes to MHL research by developing a vignette-based recognition accuracy (PDR-V) tool to assist in measuring psychological distress recognition and disorder identification. We report its internal consistency, item-level functioning, and confusion matrices and demonstrate theoretically coherent associations between PDR-V and the FFMQ facets. We also show that PDR-V scores vary in expected ways (e.g., prior psychotherapy exposure and clinical history), further supporting the external validity of this approach to measuring psychological distress recognition.

Our newly developed two-question vignette method produced transparent, performance-based evidence for psychological disorder recognition, a key component in MHL. Internally, the scored item set (classification of the vignette as psychological and identification across psychological disorders) showed exemplary internal consistency (KR-20 = 0.83), with item difficulty ranging from 0.72 to 0.95 and corrected item-total correlations spanning from 0.32 to 0.72 (median for overall PDR-V= 0.46; median for question one = 0.36; median for question two = 0.60). The physical health conditions that acted as decoys (e.g., asthma and heart disease) that were kept unscored by design functioned as a specificity/quality check (correctly classified as physical by 96% and 91.3% for heart disease and asthma; correctly classified as heart disease and asthma by 75.9% and 82.9%), mitigating concerns regarding over-pathologizing. We separated problem detection/classification (question one) from diagnostic identification accuracy (question two), distinguishing false negatives from label errors.

Content validity was supported by the current results, in which recognition rates were seemingly high in a sample of participants from English-speaking countries, with the majority of accuracy rates on the nature of the disorder (e.g., physical or psychological) falling between the ranges of 74 and 95%, with the lowest being 74% for GAD. Accurate labelling of psychological disorders was primarily between 72 and 83%, with the lowest being 72% for GAD. According to research conducted in English-speaking countries, MHL rates are seemingly increasing, with rates of recognizing it as a psychological problem typically being above 75% in Canada and Australia for depression [50,51] and 84% in Australia for schizophrenia [27]. Findings from a United States study showed that undergraduate students were able to correctly assign psychological disorders to each vignette, with 87% correctly assigning SAD and 88% correctly assigning MDD [52]. However, other studies have demonstrated differing MHL levels. Vimalanathan and Furnham’s (2019) [53] study compared MHL to health literacy and found that only 41% of their British sample could correctly identify schizophrenia (compared to 79% of our sample), and 46.8% could correctly identify bipolar disorder (compared to 79% of our sample). Therefore, MHL seems to be increasing in Western samples.

More education has previously been found to be associated with better MHL [47,48]. However, in the present study, those with education below a Bachelor’s degree scored higher on the PDR-V than those with a Bachelor’s degree or higher. The inverse relationship between education level and recognition accuracy may be best explained by the “Dilution Effect” [54]. Individuals with higher education typically possess a higher “Need for Cognition”, which corresponds to a preference for complex information processing [55]. While often advantageous, this cognitive style can be detrimental in specific diagnostic tasks. Tetlock and Boettger (1989) [56] demonstrated that individuals motivated to think complexly are paradoxically more susceptible to the dilution effect, as they attempt to integrate non-diagnostic details (e.g., a character’s job or marital status) into their judgment, which “waters down” the predictive validity of clear diagnostic cues. In contrast, participants with lower education may have employed more heuristic processing, focusing solely on the salient symptoms and thus achieving higher recognition rates for these specific vignettes. Alternatively, our grouping of education may misrepresent educational level, as one study classified high school diplomas and above as higher education [57]. Only 1.7% (*n* = 5) of our sample had an education level lower than a high school diploma, and if it was grouped differently, more education would likely result in higher recognition scores on the vignettes. Further, we did not specifically ask participants’ educational background, and there are several social sciences and humanities certificates and diplomas with high acceptance rates that revolve around mental health (e.g., Mental Health and Addictions Counselling diplomas) [58].

Consistent with previous assumptions, those with exposure to psychotherapy in the past had significantly higher total scores on the vignettes. This is likely due to the common psychotherapeutic component of psychoeducation that focuses on educating the client on their symptoms and mental health broadly [59]. The information garnered during psychoeducation may be carried after termination of psychotherapy, as one study with adolescents using an internet-delivered cognitive behavioural therapy (ICBT) program found that participants tended to remember and apply cognitive behavioural principles to present situations six months after [60]. A meta-analysis demonstrated that internet-delivered educational programs improved MHL significantly, specifically symptoms and mental health treatments [61], and a study of undergraduate students found that the strongest variant in MHL scores was whether a clinical psychology course was taken, and they were likely to be those who had been treated for a psychological disorder [8]. A similar concept to prior psychotherapy experience is having a prior mental health diagnosis. We found that higher performers on the vignettes were those with a prior mental health diagnosis, which may tie into prior psychotherapy experience, as receiving a diagnosis may warrant further intervention depending on the severity [62]. Consistent with our results, other research suggests that high performers on MHL were likely to have been diagnosed with a psychological disorder [8], and having a family history of mental illness was related to higher MHL [63], which may be due to exposure to terminology or heightened alert to psychological symptoms. Additionally, receiving a diagnosis may compel individuals to seek information to obtain a better understanding of themselves, and they are also exposed to the assessment and diagnostic processes [64].

Income and gender, despite being previously related to MHL [65,66,67,68], did not show significant group differences between low and high income and those identifying as men or women for PDR-V total scores. Income does not always imply more education, as the median salary in 2020 for Canadians with a college background was CAD 50,000 [69], and a low income for one person living in metropolitan areas is CAD 30,500 [70]. The literature consistently notes that women tend to have higher MHL [8]; however, these effects were not found in the current study, which may be due to only measuring one component of MHL. Although, we did find that those with a prior mental health diagnosis had higher correct recognition scores, which was found previously [8]. Women tend to have more diagnoses than men [71], which may explain gender differences previously. A more nuanced explanation may be that it is rather the presence of a diagnosis that impacts symptom recognition rather than gender.

Findings from prior research [18,19,20,21] suggest that there is a plausible theoretical relationship proposed between DM and MHL. However, in the present study, participants with a current mindfulness meditation practice did not score higher on the vignettes than those without a current practice. This may be due to a lack of specificity in questions, as we did not ask what type of meditation they engaged in or how often. Additionally, those practicing mindfulness are likely to have higher depressive symptoms, as it is often used as an intervention to reduce depression [72], making focusing and appropriate distress labelling more difficult and thus lowering their accuracy levels. Prior research has found that the facet of observe is particularly important in meditation [16]; however, it is associated with increases in depressive symptoms through increased rumination [73]. Therefore, it is plausible that those with mindfulness practices are higher in observe but are not necessarily high performers in MHL. Additionally, the observe facet was not correlated with symptom recognition in our results, strengthening this notion.

In the present study, describe directly related to being able to label and recognize psychological symptoms, and it is likely the most pertinent facet in symptom recognition. Describe has been previously found to be negatively related to affective symptoms [74], as a predictor of negative affect after other facets have been controlled for [75], and it is associated with one’s ability to verbally express their inner experiences, perceptions, and emotions without judgment or strong reactions [76]. Act with awareness predicted depression reductions over time [73,77], and among postpartum women, having lower depressive symptoms was associated with higher act with awareness levels [78]. These relationships with depressive and affective symptoms are important in MHL research, as having mental health diagnoses has been previously related to better MHL [8], which was also demonstrated in the present study. Of most significance is the relationship between the PDR-V and MHLS [35], a previously validated measure of MHL, which provides strong concurrent validity for the PDR-V. This correlational relationship provides evidence for PDR-V being a reliable measure of MHL, as the MHLS is widely used to measure MHL. Additionally, higher recognition scores were negatively correlated with depressive symptoms in the present study, lending further support for describe and act with awareness facets being related due to their relationships with depressive symptoms.

The PDR-V demonstrates strong internal consistency and construct validity. First, PDR-V evidenced excellent KR-20 coefficients for the scale as a whole (>0.80), indicating strong internal consistency and supporting its reliability as a cohesive measure. However, a divergence was noted between the two subcomponents. While the “diagnostic labeling” items showed excellent consistency, the “problem recognition” items were less consistent. This discrepancy is likely methodological, as the inclusion of an ambiguous “other” response option in the recognition phase may have introduced error variance, confusing participants who otherwise recognized the presence of distress. The range of item difficulty for the overall PDR-V was 0.72 to 0.95, which may indicate that items were on the easier side and may not differentiate between high and low ability. It also may explain the difference in education groups, as those with education at a Bachelor’s level and above may have overanalyzed it since its difficulty level was low.

Corrected item-total correlations generally supported the structural coherence of the PDR-V, though distinct patterns emerged across diagnoses. Schizophrenia demonstrated the strongest overall fit, likely due to the high salience of positive symptoms (e.g., hallucinations) [37,41], which are widely recognized by laypeople as markers of psychological pathology [79]. Conversely, GAD yielded the weakest correlations across both problem recognition and diagnostic labelling. This mirrors the item difficulty analysis, suggesting that the non-specific nature of GAD symptoms, which may overlap with normal stress experiences, creates greater variance in detection. Interestingly, PTSD displayed a divergent profile with weaker correlations for generic recognition but strong coherence for specific labelling. This likely reflects the disorder’s distinct etiological requirement (exposure to a traumatic event) [37,41] compared to the internalizing presentations of the other disorders. While the unique clinical features of PTSD may lower its statistical covariance with other mood disorders, its strong performance in the labelling phase supports its inclusion in the measure.

The independent *t*-tests provided strong evidence of known-groups validity for the PDR-V. As hypothesized, participants with a history of psychotherapy or a formal mental health diagnosis scored significantly higher than those without. This distinction validates the measure’s ability to detect higher levels of MHL where expected. The mechanism here is likely twofold. First, standard psychotherapeutic interventions typically incorporate a substantial component of psychoeducation regarding etiology, symptomology, and treatment options [59]. Second, individuals with a diagnosis possess prior experience with a specific disorder(s) and may be more sensitive to psychological distress and its numerous presentations, affording them a nuanced, experiential understanding of distress and the diagnostic process [80]. The fact that the PDR-V successfully differentiated these “expert” groups from the lay population strongly supports its construct validity. Not having a current mindfulness practice had higher scores on the PDR-V, contributing to its validity and reliability, as it captures the nuances of those who may have the tendency to have negative interpretations for ambiguous situations and increased rumination, also known as interpretation biases [73,81]. Since there was no relationship between GAD and PDR-V scores, low performers may have been demonstrating interpretation bias, but the PDR-V was able to capture those without mindfulness practices who were able to accurately depict the psychological disorder being represented.

The lack of correlations observed in the remaining facets (i.e., observe, non-judgment, and non-reactivity) and other variables (i.e., GAD-7, MHSAS) provides support for the reliability, convergent, and discriminant validity for the PDR-V. Honing observe requires meditative experience [16], and meditators in the present sample were small (28.4%). Observe has been found to predict increases in depression because of increased rumination [73], and the correlation between PDR-V and depressive symptoms in the present study was negative rather than positive. This provides support for PDR-V’s validity and reliability since observe was not related to PDR-V scores and has shown to be associated with an increase in depression [73], while PDR-V was found to have an inverse relationship with depression in the present study. Further, non-reactivity, non-judgment, and MHSAS were not associated with PDR-V total scores. Non-reactivity and non-judgment focus on not reacting or judging an experience, respectively [16], and MHSAS only considers attitudes towards seeking help for psychological distress. The PDR-V focuses only on the recognition and identification of the psychological disorders component of MHL, not on attitudes towards psychological disorders, distress, or treatment. The lack of relationship found between PDR-V and observe, non-reactivity, non-judgment, and MHSAS is favoured, as it reliably demonstrates that the PDR-V is only measuring symptom recognition and disorder identification.

Despite a strong negative correlation between PDR-V and depressive symptoms, this did not translate to GAD symptoms. However, it is a preferred result, as it lends support for PDR-V validity. Worry and rumination, which are characterized by repetitive negative thoughts about future and past events, respectively, are key clinical features in GAD [82,83]. Researchers have noted that the propensity to describe inner and outer experiences with heightened concerns regarding negative future outcomes may be less helpful, which translates to worry [74]. Additionally, the interpretation bias [81] is often associated with increased worry and rumination [84]. Interpretation bias [81] may negatively impact one’s ability to accurately describe and classify psychological disorders, and, therefore, the finding that GAD scores were not associated with PDR-V demonstrates that participant selection and overall accuracy may not have been impacted by interpretation biases, supporting the construct validity of the PDR-V vignettes, as almost half (42.1%) received 100% on the vignettes.

The confusion matrices presented offer critical information for refinement but also demonstrate the comorbidity between disorders that has been noted in the literature prior (e.g., MDD and GAD) [84]. This also provides further strength to the PDR-V’s reliability and validity, as these disorders are commonly comorbid with each other and often misdiagnosed [85,86]. The majority of effects observed in the present study were small to medium, which is consistent with prior work on MHL [87,88].

## 6. Strengths

The current study has several robust design features. The study’s sample size (*N* = 299) was relatively large, providing sufficient power to detect differences in our outcome variables. The case vignettes used to measure problem recognition and disorder identification were developed according to the core section of the ICD-11 (i.e., mood and psychotic disorders) [41], which is used internationally. These vignettes covered six psychological disorders and used two physical disorders to avoid priming on subsequent questions. There was consensus across three independent mental health researchers on their content validity, ensuring that the vignettes were sufficiently able to measure problem recognition and disorder identification.

## 7. Limitations and Future Directions

There are significant limitations to note regarding the PDR-V that pave the way for future research. The study took participants approximately 45 min to complete, which may have resulted in survey fatigue. Additionally, the MHLS was not randomized, which may have introduced order effects. Despite passing our attention check question, it is possible participants may have been responding more rapidly to finish the survey. Further, despite a requirement of having an English language proficiency of 6/10 or more, it is possible that those whose English is not their first language misunderstood a question [89]. Additionally, we did not measure English language proficiency and only asked participants to rate their own, introducing bias into how they report their proficiency levels [89]. Future research should examine these hypotheses in different languages to further understand DM and MHL relationships more broadly.

Since all measures were collected in a single session using self-report measures and vignette judgments, shared method variance cannot be ruled out. The PDR-V indexes were also only one of six domains of MHL; therefore, it does not assess knowledge of causes, treatment beliefs, or stigma, so generalizations to “overall MHL” are cautioned. The design was cross-sectional, thus not allowing conclusions to draw causal inferences or tease apart DM and MHL components, which is a significant limitation. Most participants were White (77.3%) and from English-speaking countries strictly, and there were no country data to report on, limiting the generalizability of the results, as several ethnicities were not represented, and we cannot view performance across countries. Mental health and MHL alike are deeply influenced by cultural factors, and the lack of representation is a significant limitation, as the nuances of culture are not captured in understanding symptom recognition and disorder identification [90]. While the use of vignette methodology likely contributed to improved ecological validity of the present study, the approach also has limitations, as we were only able to ask participants two questions after each vignette.

The present study’s sample fit criteria for a “WEIRD” sample, classified as being from Western, Educated, Industrialized, Rich, and Democratic Societies, significantly impacting generalizability [91]. However, it should be noted that the present study is meant to serve as a preliminary study to pave the way for future research to generalize across different samples. Discussion around “WEIRD” samples includes notions that generalization across all people in research is not attainable [92]. Therefore, further research is strongly encouraged to explore the PDR-V in different contexts and populations to fill the significant limitations presented in our study.

The present study’s findings, together with its limitations, highlight important avenues for future investigations. Future research should evaluate whether PDR-V scores prospectively predict real-world treatment seeking and whether changes in mindfulness levels over time correspond to changes in recognition accuracy. Of significant importance is testing whether mindfulness training improves psychological distress recognition and requires a randomized longitudinal approach. We also recommend continued psychometric work on PDR-V, including cross-cultural calibration, item revisions for diagnoses that were frequently misclassified, and counterbalancing vignette order.

## 8. Conclusions

Through demonstration of strong internal consistency and variations in PDR-V performances as a result of different demographic profiles (e.g., prior psychotherapy exposure, clinical history), the PDR-V is capable of being utilized to measure an individual’s ability to recognize psychological distress and appropriately identify the psychological disorder. The associations between FFMQ facets and MHL provide additional support, as qualities of DM, specifically the facets proposed in the FFMQ, have been theorized to be associated with better MHL. As scores on the PDR-V indicated positive relationships between describe and act with awareness facets, both of which have been linked to better recognition and description of emotions^18^ and predictors of MHL and TSAs^21^, there is further evidence for the PDR-V’s ability to accurately measure distress recognition and identification. The vignettes were also developed according to ICD criteria [41] and reviewed by mental health researchers for accuracy, strengthening their applicability internationally rather than just in North America. However, as noted previously, the present study is limited by a WEIRD sample that lacks diversity and other characteristics that would provide insight (e.g., no country data).

While the PDR-V exhibits promising psychometric properties, these are preliminary, and future research is warranted to address the significant limitations noted. However, the current PDR-V reliability and validity results support its utilization in MHL research, as it was effective in differentiating the presence of a problem, as well as providing the appropriate diagnosis. Future research should employ longitudinal designs, cross-cultural comparisons, and utilize a more diverse sample to draw further conclusions on the PDR-V’s reliability and validity as a measure for MHL.

## Figures and Tables

**Table 1:** Summary of pertinent sample demographics.

Total Sample (*n*)		N = 299
Age	M 41.04	SD 11.62
Gender	*n*	%
Cis woman	148	49.5
Cis man	145	48.5
Trans man	1	0.3
Prefer not to disclose	1	0.3
Not listed, please specify	4	1.3
Woman	1	0.3
Female	1	0.3
Man	1	0.3
Male	1	0.3
Ethnicity	*n*	%
Black	25	8.4
East Asian	11	3.7
Latinx	14	4.7
Middle Eastern	1	0.3
Southeast Asian	10	3.3
White	231	77.3
Another race category	6	2.0
Do not know	1	0.3
Marital Status	*n*	%
Single, never married	123	41.1
Married	136	45.5
Separated/divorced	35	11.7
Widowed	5	1.7
Children	*n*	%
Yes	144	48.2
No	154	51.5
Missing	1	0.3
Approx. Annual Income (CAD)	*n*	%
Unemployed/no yearly income	13	4.3
10,000–30,000	84	28.1
31,000–50,000	66	22.1
51,000–75,000	59	19.7
76,000–99,000	40	13.4
100,000 and over	34	11.4
None of the above, please specify	3	1.0
1800	1	0.3
6000	1	0.3
Prefer not to say	1	0.3
Highest Level of Education	*n*	%
No degree, certificate, or diploma. If so, indicate the last grade completed	5	1.7
9	1	0.3
10	1	0.3
11	1	0.3
12	1	0.3
Some college, no degree	1	0.3
Secondary (high) school diploma or equivalent	60	20.1
Trades certificate or diploma	14	4.7
Other non-university certificate or diploma	14	4.7
University certificate or diploma under Bachelor’s level	28	9.4
Bachelor’s degree	121	40.5
University certificate or diploma above Bachelor’s level	2	0.7
Master’s degree	45	15.1
Doctorate	6	2.0
Degree in medicine, dentistry, veterinary medicine, or optometry	4	1.3
Religion/Belief System	*n*	%
Christianity	157	52.5
Islam	3	1.0
Judaism	4	1.3
Buddhism	2	0.7
Atheism	50	16.7
Agnosticism	61	20.4
Other	22	7.4
First Language	*n*	%
English	294	98.3
German	1	0.3
Japanese	1	0.3
Lithuanian	1	0.3
Mandarin	1	0.3
Ukrainian	1	0.3
Prior Mental Health Diagnosis	*n*	%
Yes, please specify	94	31.4
No	192	64.2
Prefer not to disclose	13	4.3
Prior Psychotherapy	*n*	%
Yes	80	26.8
No	207	69.2
Prefer not to disclose	12	4.0
Current Mindfulness Meditation Practice	*n*	%
Yes	85	28.4
No	210	70.2
Prefer not to disclose	4	1.3
Mindfulness Knowledge (0–10 Scale)	*n*	%
0	15	5.0
1	40	13.4
2	29	9.7
3	26	8.7
4	23	7.7
5	41	13.7
6	36	12.0
7	36	12.0
8	23	7.7
9	9	3.0
10	21	7.0
Prior Mindfulness-Based Intervention Participation	*n*	%
Yes	49	16.4
No	244	81.6
Prefer not to disclose	6	2.0
Current Anti-Depressant Medication	*n*	%
Yes	43	14.4
No	254	84.9
Prefer not to disclose	2	0.7

**Table 2:** Kuder–Richardson Formula 20, means, standard deviations, range of item difficulty, and corrected item-total correlations for PDR-V total score, PDR-V question 1, and PDR-V question 2.

Psychological Disorder				Question 1	Question 2
	M (SD)	KR-20	Range of Item Difficulty	Corrected Item-Total Correlations	Corrected Item-Total Correlations
PDR-V total MDD	10.04(2.57)	0.83	0.72–0.95	0.44	0.61
SAD				0.39	0.46
GAD				0.37	0.50
PTSD				0.32	0.71
Schizophrenia				0.45	0.72
Bipolar				0.34	0.59
**Psychological** **Disorder**	**M (SD)**	**KR-20**	**Range of Item Difficulty**	**Corrected Item-Total Correlations**	**Proportion Correct**
PDR-V Q1	5.32 (1.05)	0.58	0.74–0.95		
MDD				0.36	0.84
SAD				0.46	0.94
GAD				0.26	0.74
PTSD				0.26	0.92
Schizophrenia				0.39	0.94
Bipolar				0.35	0.95
**Psychological Disorder**	**M (SD)**	**KR-20**	**Range of Item Difficulty**	**Corrected Item-Total Correlations**	**Proportion Correct**
PDR-V Q2	4.72 (1.80)	0.83	0.72–0.83		
MDD				0.57	0.80
SAD				0.45	0.83
GAD				0.46	0.72
PTSD				0.74	0.80
Schizophrenia				0.76	0.79
Bipolar				0.62	0.78

Note. All values are rounded up to one decimal place. PDR-V = Psychological Disorder Recognition Vignette; MDD = Major Depressive Disorder; SAD = social anxiety disorder; GAD = generalized anxiety disorder; PTSD = post-traumatic stress disorder.

**Table 3:** (**a**) Confusion matrix (true problem × selected problem) for question one (i.e., is this a psychological, physical, or other problem). (**b**) Confusion matrix (true diagnosis × selected diagnosis) for question two (i.e., what condition is it).

**(a)**														
True↓/Pred⟶			Psychological N (%)		Physical N (%)			Other N (%)				Total N (%)		
MDD			250 (15.7)		45 (25.7)			4 (14.8)				299 (16.7)		
SAD			281 (17.7)		15 (8.6)			3 (11.1)				299 (16.7)		
GAD			222 (13.9)		62 (35.4)			15 (55.6)				299 (16.7)		
PTSD			274 (17.2)		22 (12.6)			3 (11.1)				299 (16.7)		
Schizophrenia			281 (17.7)		17 (9.7)			1 (3.7)				299 (16.7)		
Bipolar			284 (17.8)		14 (8.0)			1 (3.7)				299 (16.7)		
Total			1592 (100)		175 (100)			27 (100)				1794 (100)		
**(b)**														
**True↓/Pred⟶**	**MDD** **N (%)**	**GAD** **N (%)**	**SAD** **N (%)**	**Bipolar** **N (%)**	**PTSD** **N (%)**	**Schizophrenia** **N (%)**	**Asthma** **N (%)**	**Heart Disease** **N (%)**	**Cancer** **N (%)**	**Diabetes** **N (%)**	**Stroke** **N (%)**	**Arthritis** **N (%)**	**Other** **N (%)**	**Total** **N (%)**
MDD	238 (73.0)	25 (7.9)	8 (2.7)	3 (1.2)	1 (0.4)	1 (0.4)	1 (10.0)	2 (16.7)	1 (33.3)	2 (14.3)	6 (75.0)	2 (33.3)	9 (23.7)	299 (16.7)
SAD	14 (4.3)	26 (8.2)	248 (82.7)	2 (0.8)	1 (0.4)	2 (0.8)	1 (10.0)	1 (8.3)	0 (0)	1 (7.1)	1 (12.5)	1 (16.7)	1 (2.6)	299 (16.7)
GAD	20 (6.1)	216 (67.9)	15 (5.0)	5 (2.0)	2 (0.8)	3 (1.2)	4 (40.0)	3 (25.0)	2 (66.7)	10 (71.4)	0 (0)	2 (33.3)	17 (44.7)	299 (16.7)
PTSD	12 (3.7)	23 (7.2)	11 (3.7)	4 (1.6)	238 (95.6)	1 (0.4)	1 (10.0)	3 (25.0)	0 (0)	1 (7.1)	1 (12.5)	0 (0)	4 (10.5)	299 (16.7)
Schizophrenia	22 (6.7)	9 (2.8)	11 (3.7)	8 (3.1)	4 (1.6)	237 (93.3)	2 (20.0)	2 (16.7)	0 (0)	0 (0)	0 (0)	1 (16.7)	3 (7.9)	299 (16.7)
Bipolar	20 (6.1)	19 (6.0)	7 (2.3)	234 (91.4)	3 (1.2)	10 (3.9)	1 (10.0)	1 (8.3)	0 (0)	0 (0)	0 (0)	0 (0)	4 (10.5)	299 (16.7)
Total	326 (100)	318 (100)	300 (100)	256 (100)	249 (100)	254 (100)	10 (100)	12 (100)	3 (100)	14 (100)	8 (100)	6 (100)	38 (100)	1794 (100)

Note: MDD = Major Depressive Disorder; SAD = social anxiety disorder; GAD = generalized anxiety disorder; PTSD = post-traumatic stress disorder; rows = true (correct diagnoses); columns = participant selections; diagonal cells = % correctly identified; off-diagonal cells = all specific misclassifications; each row = 100% of responses for that true disorder. Arrows indicate corresponding row or column for true problem/diagnosis versus predicted (selected) problem/diagnosis.

**Table 4:** Problem recognition (PDR-V) scores for each psychological disorder vignette, overall score, and frequencies for total scores.

Psychological Disorder	Question 1		Question 2	
	Correct *n* (%)		Correct *n* (%)	
MDD	250 (83.6)		238 (79.6)	
SAD	281 (94.0)		248 (82.9)	
GAD	222 (74.2)		216 (72.2)	
PTSD	274 (91.6)		238 (79.6)	
Schizophrenia	281 (94.0)		237 (79.3)	
Bipolar Disorder	284 (95.0)		234 (78.3)	
	***M***	**SD**		***n*** **(%)**
PDR-V Total	10.04	2.57		
12/12				126 (42.1)
11/12				59 (19.7)
10/12				29 (9.7)
9/12				21 (7.0)
8/12				12 (4.0)
7/12				9 (3.0)
6/12				16 (5.4)
5/12				10 (3.3)
4/12				8 (2.7)
3/12				8 (2.7)
2/12				1 (0.3)

Note: All values rounded up to one decimal place. PDR-V = Psychological Disorder Recognition Vignette; MDD = Major Depressive Disorder; SAD = social anxiety disorder; GAD = generalized anxiety disorder; PTSD = post-traumatic stress disorder.

**Table 5:** Spearman correlation coefficients between study variables.

Variable	1	2	3	4	5	6	7	8	9	10
1. PDR-V	-	0.052	0.191 *	0.212 *	−0.118 **	0.112	−0.176 *	−0.110	0.102	0.545 *
2. FFMQ_O	-	-	0.319 ***	0.185 ***	0.175 ***	0.00	−0.082	−0.084	0.143 **	0.072
3. FFMQ_D	-	-	-	0.511 ***	0.325 ***	0.428 ***	−0.489 ***	−0.506 ***	0.220 ***	0.208 ***
4. FFMQ_AA	-	-	-	-	0.250 ***	0.516 ***	−0.642 ***	−0.618 **	0.268 ***	0.118 **
5. FFMQ_NR	-	-	-	-	-	0.292 ***	−0.380 ***	−0.454 ***	0.179 ***	−0.071
6. FFMQ_NJ	-	-	-	-	-	-	−0.489 ***	−0.546 ***	0.133 **	0.124 **
7. PHQ-9	-	-	-	-	-	-	-	0.830 ***	−0.363 ***	−0.170 ***
8. GAD-7	-	-	-	-	-	-	-	-	−0.345 ***	−0.147 **
9. MHSAS	-	-	-	-	-	-	-	-	-	0.403 ***
10. MHLS	-	-	-	-	-	-	-	-	-	-

Note: PDR-V = Psychological Disorder Recognition—Vignette; FFMQ_O = Five Facet Mindfulness Questionnaire with Observe facet; FFMQ_D = Five Facet Mindfulness Questionnaire with Describe facet; FFMQ_AA = Five Facet Mindfulness Questionnaire Act with Awareness facet; FFMQ_NR = Five Facet Mindfulness Questionnaire with Non-reactivity facet; FFMQ_NJ = Five Facet Mindfulness Questionnaire with Non-judgment facet; PHQ-9 = Patient Health Questionnaire-9; GAD-7 = generalized anxiety disorder-7; MHSAS = Mental Help Seeking Attitudes Scale; MHLS = Mental Health Literacy Scale. All values are rounded to the nearest decimal place. * = Significant at Holm-corrected *p* < 0.05. ** = Significant before Holm-corrected at *p* < 0.05; *** = Significant before Holm-corrected at *p* < 0.01; Bold: Significant Holm-corrected correlations.

**Table 6:** Independent sample *t*-tests for education level, income, psychotherapy exposure, prior mental health diagnosis, and gender.

Variable	N	*M* (SD)	*F*	*Sig*	*t*	*df*	*p*	*d*	Lower	Upper
**Education**								0.67	0.432	0.906
Under Bachelor’s	121	11.02 (1.51)								
Bachelor’s or higher	178	9.38 (2.91)								
**Equal Variances Assumed**			76.871	<0.001	5.683	297	<0.001			
**Equal Variances Not Assumed**					6.345	280.327	<0.001 *			
**Income**								0.258	0.027	0.487
≤50,000 CAD	163	10.36 (2.31)								
≥51,000 CAD	133	9.70 (2.81)								
**Equal Variances Assumed**			10.878	0.001	2.205	294	0.028			
**Equal Variances Not Assumed**					2.161	254.242	0.032			
**Psychotherapy exposure**								−0.591	−0.853	−0.328
Yes	80	11.09 (1.74)								
No	207	9.60 (2.75)								
**Equal Variances Assumed**			34.348	<0.001	−4.489	285	<0.001			
**Equal Variances Not Assumed**					−5.445	225.122	<0.001 *			
**Prior mental health diagnosis**								−0.518	−0.768	−0.267
Yes	94	10.91 (1.79)								
No	192	9.61 (2.79)								
**Equal Variances Assumed**			35.436	<0.001	−4.114	284	<0.001			
**Equal Variances Not Assumed**					−4.751	263.873	<0.001 *			
**Current mindfulness practice**								0.574	0.318	0.830
Yes	85	9.02 (3.12)								
No	210	10.45 (2.18)								
**Equal Variances Assumed**			35.632	<0.001	4.467	293	<0.001			
**Equal Variances Not Assumed**					3.854	118.546	<0.001 *			
**Gender**								0.057	−0.170	0.284
Woman	150	10.11 (2.72)								
Man	148	9.97 (2.42)								
**Equal Variances Assumed**			2.045	0.154	0.493	296	0.622			
**Equal Variances Not Assumed**					0.494	292.859	0.622			

Note: All values are rounded to the nearest decimal place. * = Significant at Holm-corrected *p* < 0.05. Bold: Significant correlations.

## Data Availability

The de-identified data is available on the last author’s Open Science Framework: https://doi.org/10.17605/OSF.IO/QXPB3 accessed on 21 September 2024.

## Supplementary Materials

The following supporting information can be downloaded at https://www.mdpi.com/article/10.3390/ijerph23010031/s1. Supporting information includes the PDR-V (S1) and gender specificity analyses (S2).

## References

[1] World Health Organization (2019). Mental Health. https://www.who.int/health-topics/mental-health#tab=tab_2.

[2] Kohn R., Ali A.A., Puac-Polanco V., Figueroa C., López-Soto V., Morgan K., Saldivia S., Vicente B. (2018). Mental health in the Americas: An overview of the treatment gap. Rev. Panam. Salud Publica.

[3] Conroy J., Lin L., Ghaness A. (2020). Why people aren’t getting the care they need. Monit. Psychol..

[4] Canadian Mental Health Association (2008). The Relationship Between Mental Health, Mental Illness, and Chronic Physical Conditions. https://ontario.cmha.ca/documents/the-relationship-between-mental-health-mental-illness-and-chronic-physical-conditions/.

[5] Jorm A.F. (2000). Mental health literacy: Public knowledge and beliefs about mental disorders. Br. J. Psychiatry.

[6] Curran T., Ito-Jaeger S., Perez Vallejos E., Crawford P. (2023). “What’s up everyone?”: The effectiveness of a digital media mental health literacy campaign for young people. J. Ment. Health.

[7] Noroozi A., Khademolhosseini F., Lari H., Tahmasebi R. (2018). The mediator role of mental health literacy in the relationship between demographic variables and health-promoting behaviours. Iran J. Psychiatry Behav. Sci..

[8] Miles R., Rabin L., Krishnan A., Grandoit E., Kloskowski K. (2020). Mental health literacy in a diverse sample of undergraduate students: Demographic, psychological, and academic correlates. BMC Public Health.

[9] Protheroe J., Whittle R., Bartlam B., Estacio E.V., Clark L., Kurth J. (2017). Health literacy, associated lifestyle and demographic factors in adult population of an English city: A cross-sectional survey. Health Expect..

[10] Kabat-Zinn J. (2003). Mindfulness-based interventions in context: Past, present, and future. Clin. Psychol. Sci. Pract..

[11] Bishop S.R., Lau M., Shapiro S., Carlson L., Anderson N.D., Carmody J., Segal Z.V., Abbey S., Speca M., Velting D. (2004). Mindfulness: A proposed operational definition. CPSP.

[12] Kabat-Zinn J. (1990). Full Catastrophe Living: Using the Wisdom of Your Body and Mind to Face Stress, Pain, and Illness.

[13] Schmidt S. (2011). Mindfulness in east and west—Is it the same?. Neuroscience, Consciousness and Spirituality.

[14] Tomlinson E.R., Yousaf O., Vittersø A.D., Jones L. (2018). Dispositional mindfulness and psychological health: A systematic review. Mindfulness.

[15] Chiesa A. (2013). The difficulty of defining mindfulness: Current thought and critical issues. Mindfulness.

[16] Baer R.A., Smith G.T., Hopkins J., Krietemeyer J., Toney L. (2006). Using self-report assessment methods to explore facets of mindfulness. Assessment.

[17] Bodhi B. (2000). A Comprehensive Manual of Abhidhamma: The Philosophical Psychology of Buddhism.

[18] Karl J.A., Lassen E.R., Solem S., Fischer R. (2024). Between can’t and won’t: The relationship between trait mindfulness, stoic ideology, and alexithymia in Norway and New Zealand. Mindfulness.

[19] Lo C.C., Chen I.C., Ho W.S., Cheng Y.C. (2023). A sequential meditation model of perceived social support, mindfulness, perceived hope, and mental health literacy: An empirical study on Taiwanese university of students. Acta Psychol..

[20] Daya Z., Hearn J.H. (2018). Mindfulness interventions in medical education: A systematic review of their impact on medical student stress, depression, fatigue and burnout. Med. Teach..

[21] Gerbeza M., Dabek K., Lockinger K., Wilkens I.M., Loarca-Rodriguez M., Grogan K., Beshai S. (2025). Beyond the unitary: How facets of dispositional mindfulness predict, moderate, and mediate mental health literacy and treatment seeking attitudes. Healthcare.

[22] Ajzen I. (1991). The theory of planned behavior. Organ. Behav. Hum. Decis. Process..

[23] Bowlin S.L., Baer R.A. (2012). Relationships between mindfulness, self-control, and psychological functioning. Personal. Individ. Differ..

[24] Hudson N.W., Anusic I., Lucas R.E., Donnellan M.B. (2020). Comparing the reliability and validity of global self-report measures of subjective well-being to experiential day reconstruction measures. Assessment.

[25] Paulhus D.L., Vazire S. (2007). The self-report method. Handb. Res. Methods Personal. Psychol..

[26] Finch J. (1987). Research note: The vignette technique in survey research. Sociology.

[27] Jorm A.F., Korten A.E., Jacomb P.A., Christensen H., Rodgers B., Pollitt P. (1997). “Mental health literacy”: A survey of the public’s ability to recognise mental disorders and their beliefs about the effectiveness of treatment. Med. J. Aust..

[28] Blum R.W., Sheehy G., Li M., Basu S., Gibaly O.E., Kayembe P., Zuo X., Ortiz J., Chan K.S., Moreau C. (2019). Measuring young adolescent perceptions of relationships: A vignette-based approach to exploring gender equality. PLoS ONE.

[29] Erfanian F., Roudsari R.L., Haidari A., Bahmani M.N.D. (2019). A narrative on using vignettes: Its advantages and drawbacks. JMRH.

[30] Marshall J.M., Dunstan D.A. (2011). Mental health literacy of Australian rural adolescents: An analysis using vignettes and short films. Aust. Psychol..

[31] Gerbeza M. (2023). The Associations of Dispositional Mindfulness with Recognition of Psychological Disorders and Willingness to Seek Help. Ph.D. Thesis.

[32] Litman L., Robinson J., Abberbock T. (2017). TurkPrime.com: A versatile crowdsourcing data acquisition platform for the behavioral sciences. Behav. Res. Methods.

[33] Beshai S., Prentice J.L., Huang V. (2018). Building blocks of emotional flexibility: Trait mindfulness and self-compassion are associated with positive and negative mood shifts. Mindfulness.

[34] Chandler J., Shapiro D. (2016). Conducting clinical research using crowdsourced convenience samples. Annu. Rev. Clin. Psychol..

[35] O’Connor M., Casey L. (2015). The mental health literacy scale (MHLS): A new scale-based measure of mental health literacy. Psychiatry Res..

[36] Kroenke K., Spitzer R.L., Williams J.B. (2001). The PHQ-9: Validity of a brief depression severity measure. J. Gen. Intern. Med..

[37] American Psychiatric Association (2022). Diagnostic and Statistical Manual of Mental Disorders.

[38] Spitzer R.L., Kroenke K., Williams J.B., Löwe B. (2006). A brief measure for assessing generalized anxiety disorder. Arch. Intern. Med..

[39] Bohlmeijer E., Ten Klooster P.M., Fledderus M., Veehof M., Baer R. (2011). Psychometric properties of the five facet mindfulness questionnaire in depressed adults and development of a short form. Assessment.

[40] Hammer J.H., Parent M.C., Spiker D.A. (2018). Mental Help Seeking Attitudes Scale (MHSAS): Development, reliability, validity, and comparison with the ATSSPH-SF and IASMHS-PO. J. Couns. Psychol..

[41] World Health Organization (2021). International Statistical Classification of Diseases and Related Health Problems.

[42] Newman L.S., Tan M., Caldwell T.L., Duff K.J., Winer E.S. (2018). Name norms: A guide to casting your next experiment. PSPB.

[43] Solmi M., Radua J., Olivola M., Croce E., Soardo L., Salazar de Pablo G., Il Shin J., Kirkbride J.B., Jones P., Kim J.H. (2022). Age at onset of mental disorders worldwide: Large-scale meta-analysis of 192 epidemiological studies. Mol. Psychiatry.

[44] Salimuddin S. (2023). Problem Recognition and Treatment Recommendations of Somatic and Cognitive-Affective Presentations of Depression and Generalized Anxiety. Master’s Thesis.

[45] Wijeratne C., Harris P. (2009). Late life depression and dementia: A mental health literacy survey of Australian general practitioners. Int. Psychogeriatr..

[46] Uyanah D.A., Nsikhe U.I. (2023). The theoretical and empirical equivalence of Cronbach alpha and Kuder-Richardson Formular-20 reliability coefficients. IRJIET.

[47] Lee H.Y., Hwang J., Ball J.G., Lee J., Albright D.L. (2019). Is health literacy associated with mental health literacy? Findings from Mental Health Literacy Scale. Perspect. Psychiatr. Care.

[48] Schröder R., Hamer T., Suhr R., König L. (2025). Attitudes toward psychotherapeutic treatment and health literacy in a large sample of the general population in Germany: Cross-sectional study. JMIR Public Health Surveill..

[49] Niemeyer H., Knaevelsrud C. (2023). Socioeconomic status and access to psychotherapy. J. Clin. Psychol..

[50] Jorm A.F., Nakane Y., Christensen H., Yoshioka K., Griffiths K.M., Wata Y. (2005). Public beliefs about treatment and outcome of mental disorders: A comparison of Australia and Japan. BMC Med..

[51] Kitchener B.A., Jorm A.F. (2002). Mental health first aid training for the public: Evaluation of effects on knowledge, attitudes and helping behavior. BMC Psychiatry.

[52] Coles M.E., Coleman S.L. (2010). Barriers to treatment seeking for anxiety disorders: Initial data on the role of mental health literacy. Depress. Anxiety.

[53] Vimalanathan A., Furnham A. (2019). Comparing physical and mental health literacy. J. Ment. Health.

[54] Nisbett R.E., Zukier H., Lemley R.E. (1981). The dilution effect: Nondiagnostic information weakens the implications of diagnostic information. Cogn. Psychol..

[55] Cacioppo J.T., Petty R.E. (1982). The need for cognition. J. Pers. Soc. Psychol..

[56] Tetlock P.E., Boettger R. (1989). Accountability: A social magnifier of the dilution effect. J. Pers. Soc. Psychol..

[57] Assaf E., Nehme M., Eid C., Chelala H., Nohra D. (2025). Associations between socio-economic factors, mental health knowledge, and psychological distress among Lebanese adults: A cross-sectional study. BMC Psychol..

[58] Saskatchewan Polytechnic School of Health Sciences (2025). Mental Health and Addictions Counselling. https://saskpolytech.ca/programs-and-courses/programs/Mental-Health-and-Addictions-Counselling.aspx.

[59] Lukens E.P., McFarlane W.R. (2004). Psychoeducation as evidence-based practice: Considerations for practice, research, and policy. BTIC.

[60] Berg M., Malmquist A., Rozental A., Topooco N., Andersson G. (2020). Knowledge gain and usage of knowledge learned during internet-based CBT treatment for adolescent depression—A qualitative study. BMC Psychiatry.

[61] Nazari A., Garmaroudi G., Foroushani A.R., Hosseinnia M. (2023). The effect of web-based educational interventions on mental health literacy, stigma and help-seeking intentions/attitudes in young people: Systematic review and meta-analysis. BMC Psychiatry.

[62] Elliot K.P., Westmacott R., Hunsley J., Rumstein-McKean R., Best M. (2015). The process of seeking psychotherapy and its impact on therapy expectations and experiences. Clin. Psychol. Psychother..

[63] Beyazel E., Kendirkıran G. (2025). Mental health literacy level and barriers to seeking mental health counseling in adults. J. Psychiatr. Nurs..

[64] Perkins A., Ridler J., Browes D., Peryer G., Notley C., Hackmann C. (2018). Experiencing mental health diagnosis: A systematic review of service user, clinician, and carer perspectives across clinical settings. Lancet Psychiatry.

[65] Cotton S.M., Wright A., Harris M.G., Jorm A.F., McGorry P.D. (2006). Influence of gender on mental health literacy in young Australians. Aust. N. Z. J. Psychiatry.

[66] Hadjimina E., Furnham A. (2017). Influence of age and gender on mental health literacy of anxiety disorders. Psychiatry Res..

[67] Wong D.F.K., Lam A.Y.K., Poon A., Chow A.Y.M. (2011). Gender differences in mental health literacy among Chinese-speaking Australians in Melbourne, Australia. Int. J. Soc. Psychiatry.

[68] Yu Y., Liu Z., Liu X., Liu H., Yang J.P., Zhou L., Xiao S. (2015). Assessment of mental health literacy using a multifaceted measure among a Chinese rural population. BMJ Open.

[69] Statistics Canada (2025). Estimated Gross Annual Earnings of Postsecondary Graduates Working Full Time at Interview, by Province of Study, Level of Study and Gender. https://www150.statcan.gc.ca/t1/tbl1/en/tv.action?pid=3710003401.

[70] Statistics Canada (2025). Low Income-Cutoffs (LICOs) Before and After Tax by Community Size and Family Size, in Current Dollars. https://www150.statcan.gc.ca/t1/tbl1/en/tv.action?pid=1110024101&pickMembers%5B0%5D=2.2&cubeTimeFrame.startYear=2023&cubeTimeFrame.endYear=2023&referencePeriods=20230101%2C20230101.

[71] Klose M., Jacobi F. (2004). Can gender differences in the prevalence of mental disorders be explained by sociodemographic factors?. Arch. Womens Ment. Health.

[72] Schreiner I., Malcolm J.P. (2008). The benefits of mindfulness meditation: Changes in emotional states of depression, anxiety, and stress. Behav. Change.

[73] Royuela-Colomer E., Calvete E. (2016). Mindfulness facets and depression in adolescents: Rumination as a mediator. Mindfulness.

[74] Carpenter J.K., Conroy K., Gomez A.F., Curren L.C., Hofmann S.G. (2019). The relationship between trait mindfulness and affective symptoms: A meta-analysis of the Five Facet Mindfulness Questionnaire (FFMQ). Clin. Psychol. Rev..

[75] Mandal S.P., Arya Y.K., Pandey R. (2012). Mental health and mindfulness: Mediational role of positive and negative affect. SIS J. Porj. Psy. Ment. Health.

[76] Gao T.Y., Huang F.H., Liu T., Sum R.K.W., Liu J.D., Tang D., Cai D.Y., Jiang Z.K.J., Ma R.S. (2024). The role of physical literacy and mindfulness on health-related quality of life among college students during the COVID-19 pandemic. Sci. Rep..

[77] Cash M., Whittingham K. (2010). What facets of mindfulness contribute to psychological well-being and depressive, anxious, and stress-related symptomatology?. Mindfulness.

[78] Hulsbosch L.P., van de Poel E., Nyklíček I., Boekhorst M.G.B.M. (2023). Trait mindfulness facets as a protective factor for the development of postpartum depressive symptoms. J. Psychiatr. Res..

[79] Erritty P., Wydell T.N. (2013). Are lay people good at recognising the symptoms of schizophrenia?. PLoS ONE.

[80] Bohman B. (2023). Clinicians’ perceptions and practices of diagnostic assessment in psychiatric services. BMC Psychiatry.

[81] Hirsch C.R., Mathews A. (2012). A cognitive model of pathological worry. Behav. Res. Ther..

[82] Borkovec T.D., Inz J. (1990). The nature of worry in generalized anxiety disorder: A predominance of thought activity. Behav. Res. Ther.

[83] van der Heiden C., Methorst G., Muris P., van der Molen H.T. (2011). Generalized anxiety disorder: Clinical presentation, diagnostic features, and guidelines for clinical practice. J. Clin. Psychol..

[84] Krahé C., Whyte J., Bridge L., Loizou S., Hirsch C.R. (2019). Are different forms of repetitive negative thinking associated with interpretation bias in generalized anxiety disorder and depression?. Clin. Psychol. Sci..

[85] Kaufman J., Charney D. (2000). Comorbidity of mood and anxiety disorders. Depress. Anxiety.

[86] Kasper S. (2006). Anxiety disorders: Under-diagnosed and insufficiently treated. Int. J. Psychiatry Clin. Pract..

[87] Sun G., Wang C., Zhang J. (2025). Effectiveness of mental health literacy interventions for adolescents: A systematic review and meta-analysis. SAGE Open.

[88] Yeo G., Reich S.M., Liaw N.A., Chia E.Y.M. (2024). The effect of digital mental health literacy interventions on mental health: Systematic review and meta-analysis. J. Med. Internet Res..

[89] Park H., Sha M.M., Willis G. (2016). Influence of English-language proficiency on the cognitive processing of survey questions. Field Methods.

[90] Hwang W., Myers H.F., Abe-Kim J., Ting J.Y. (2008). A conceptual paradigm for understanding culture’s impact on mental health: The cultural influences on mental health (CIMH) model. Clin. Psychol. Rev..

[91] Henrich J., Heine S.J., Norenzayan A. (2010). The weirdest people in the world?. Behav. Brain Sci..

[92] Sherman J.W. (2025). There is nothing WEIRD about basic research: The critical role of convenience samples in psychological science. Am. Psychol..

