# Supplementary material for: A Vignette-Based Measure of Mental Health Literacy (PDR-V): Reliability, Validity, and Mindfulness Associations in a Cross-Sectional Sample
Source: Int J Environ Res Public Health. 2025 Dec 24;23(1):31. doi: 10.3390/ijerph23010031 (PMC12841322; doi:10.3390/ijerph23010031)
Supplement: Supplementary file 1 [file ijerph-23-00031-s001.zip › Supplemental Files S1 and S2.pdf]

Supplemental File S1: Case Vignettes

Asthma: Mark is 35 years old. Mark has recently been having bouts of coughing at night and has not been sleeping well. He finds that these problems get worse during challenging situations, in the spring and fall, and during strenuous sports activities. This has been noticeable to his parents, professors and coaches. Mark used to enjoy playing soccer but recently gave it up because of these problems. Mark feels badly about his breathing problems, which seem to be getting worse. Besides a history of breathing problems, Mark has not had any major health conditions in the past. He is currently not on medication and does not drink or use any substance.

MDD: Michelle is 52 years old. Michelle has been feeling unusually down on most days for the last few weeks. Michelle feels like she has no energy and like she is tired all the time. She has been sleeping excessively, especially during the day, and has gained a significant amount of weight in the last few weeks. Further, she has been moving and speaking more slowly than usual, much to the concern of her loved ones. As a consequence of these developments, Michelle has been falling behind at work and missing deadlines. Michelle rarely drinks and has not had any major health conditions in the past. She is also currently not on medication.

SAD: Emma is 34 years old. For the past six months, Emma has been experiencing excessive and consistent worrying during social situations such as conversations with others and eating at restaurants. She worries that she will act in a way that the people around her will judge her and her actions, and that this will result in them rejecting her or gossiping about her. Emma has started to avoid these situations, including attending school. Her avoidance of school has caused her to fall behind in her schoolwork, and her social network is getting narrower because she is avoiding social situations altogether. Additionally, Emma finds herself trembling and

blushing excessively during these social situations. Emma is not on any medication and does not use any substances. She has no history of any major health conditions.

GAD: Anna is 30 years old. For the last few months, Anna has been worrying about a number of things such as her job, her relationship, the health of her parents, getting to places on time and making sure she is on top of her day-to-day household chores. Anna has also been experiencing gastrointestinal symptoms such as nausea. Additionally, she has dry mouth, and her muscles have been feeling tense. Her sleep has also been unsatisfying lately as she does not feel rested after sleep. Anna has not been functioning at her usual capacity at work because of these changes. Anna does not use any substance or medication. She does not have a history of any major health condition.

Caroline: Caroline is 43 years old. About a year ago, she was in a car accident that left her with several injuries (e.g., fractured rib; broken leg). For the past few months, she has been experiencing nightmares of the car accident, and these nightmares leave her waking up terrified, sweaty, and shaky. Every time she is in a moving car, she relives details of the accident. As a result, she has stopped getting into cars and avoids talking about the accident. Additionally, Caroline also feels like she is constantly on guard, and has become easily startled by minor things, such as sudden and unexpected sounds. Caroline does not use any substance or medication. The only major health conditions she has suffered with were injuries related to the accident, which have since healed.

Schizophrenia: Noah is 33 years old. For the past few months, he has been hearing voices around him even when he is completely alone. He can no longer find the motivation to complete any task, including simple ones (e.g., keeping up with his hygiene). Noah feels as if these voices are controlling his thoughts and actions, and that everyone around him can read his

thoughts. Noah has begun to talk back to the voices, alarming those around him. As a result of these changes, he has dropped out of university and ignores his friends and family. Noah does not use any substances or medication. He does not have a history of any major health condition.

Bipolar: William is 47 years old. For the past two weeks, William has been experiencing rapid, severe, and consistent shifts in his emotions throughout the day. On some days, William feels severely upset and hopeless, and on other days he feels energetic, distracted, and impulsive. When William is feeling energetic, distracted, and impulsive, he often feels a higher sense of self-esteem and tells his wife about the many different ideas he has, even if they are not logical and may be reckless. During these euphoric states, William does not sleep. William does not use any substance or medication. He does not have a history of any major health condition.

Heart disease: Nicholas is 55 years old. Recently, his doctor has asked him to lose weight, to stop eating fatty food, and to stop smoking because his cholesterol and blood sugar levels were high. Two weeks ago, Nicholas started to get chest pains and to become short of breath whenever he walked too fast or up the stairs. The chest pain is now coming on even when he is resting. He has had high blood pressure for many years, and is on blood pressure medication. Nicholas does not drink, and does not have any history of any major health conditions other than his high blood pressure.

Supplemental File S2: Specificity analyses

Supplementary Table S1. Specificity analyses for independent sample t-tests for gender for those identifying as trans man and "open response" options not included.

**Variable**	**N**	***M* (SD)**	***F***	***Sig***	***t***	***df***	***p***	***d***	**Lower**	**Upper**
**Gender**								.063	-.166	.292
Woman	148	10.10(2.73)								
Man	145	9.94(2.43)								
Equal Variances Assumed			1.981	.160	.541	291	.589			
Equal Variances Not Assumed					.541	288.329	.589			
